# Trauma-Induced Unilateral Progression of Sclerosis in a Patient With Limited Cutaneous Systemic Sclerosis

**DOI:** 10.7759/cureus.90182

**Published:** 2025-08-15

**Authors:** Claudia Lee, Neha Garg, Stephanie Mengden-Koon, Nicole Fett

**Affiliations:** 1 Dermatology, Oregon Health and Science University, Portland, USA; 2 Rheumatology, Northwest Rheumatology Associates, Portland, USA

**Keywords:** pathogenesis, scleroderma, systemic scleroderma, trauma induced, unilateral progression, unilateral sclerosis

## Abstract

Systemic sclerosis is an autoimmune connective tissue disease, which is characterized by immune dysregulation and progressive sclerosis of the skin with variable internal organ involvement. Exogenous triggers such as physical trauma are documented as a potential precipitating factor for systemic sclerosis; however, the condition typically presents with bilateral skin changes. Unilateral progression of systemic sclerosis is an extremely rare phenomenon, and the role injury and/or immobilization plays in provoking this presentation requires further elucidation. A 63-year-old female with an 11-year history of limited cutaneous systemic sclerosis presents with rapidly progressive sclerosis confined to her right hand and wrist following a radial fracture and six months of immobilization. Examination revealed increased swelling and stiffness, considerably decreased range of motion, and progressive sclerodactyly of all five digits. MRI, blood cultures, and venous ultrasound were unremarkable. A punch biopsy was performed, and the histologic findings were consistent with scleroderma. We report a unique case of unilateral progression of limited cutaneous systemic sclerosis after injury and immobilization, which highlights the potential role of trauma and immobilization in the pathogenesis of systemic sclerosis.

## Introduction

Systemic sclerosis (SScl) is a rare autoimmune disease characterized by widespread vascular dysfunction and sclerosis of the skin and internal organs. The pathogenesis is complex and remains incompletely understood; however, it is believed that abnormal immune activation and vascular insult lead to dysregulation of fibroblasts and subsequent overproduction and deposition of collagen [1]. Several environmental triggers have been associated with inducing SScl, including infectious and chemical agents and mechanical injury [1]. Typically, these patients present with bilateral Raynaud’s phenomenon (RP) and skin thickening that begins in the distal extremities and progresses proximally and symmetrically. Unilateral progression of signs of SScl is an extremely rare phenomenon. Four cases have been reported following mechanical injury [2-4], suggesting a potential correlation between the pathogenesis of SScl and the underlying mechanisms associated with these physical effects. Due to the scarcity of cases, our understanding of the role injury plays in provoking unilateral symptoms of SScl remains limited.

## Case presentation

We report a case of rapidly progressive unilateral hand sclerosis in a 63-year-old female with a history of limited cutaneous systemic sclerosis (LcSScl). The diagnosis of LcSScl was made 11 years prior based on Raynaud's phenomenon (RP), digital ulcerations, nailfold capillary dilation, mild bilateral sclerodactyly, digital calcinosis cutis, and positive antinuclear and centromere antibodies. In the preceding 11 years, there was no progression of cutaneous sclerosis, without any immunosuppressive treatment. 

**Figure 1:**
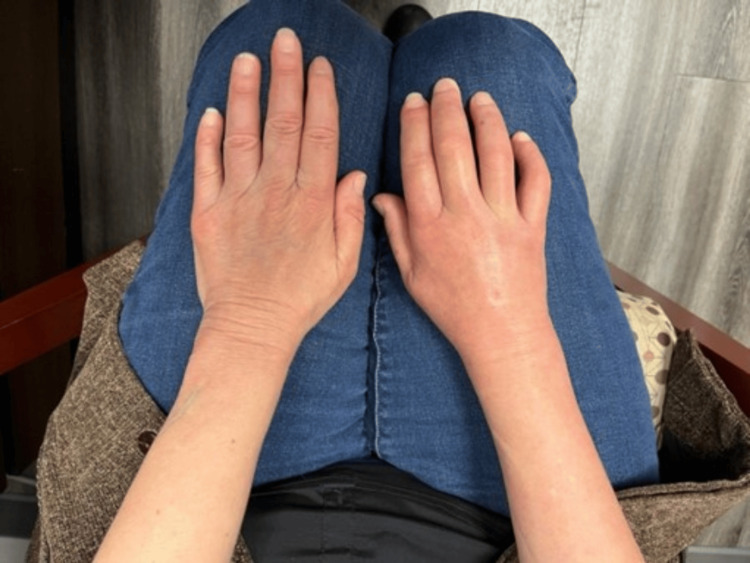
Stark scleroderma seen unilaterally on the right hand The right hand demonstrates significant swelling, taut shiny skin, with loss of skin wrinkles compared to the left hand. Early flexion contractures of the right digits are appreciated.

The patient presented with persistent right-hand and wrist swelling with decreased mobility, six months after experiencing a ground-level fall that resulted in an intra-articular radius fracture requiring immobilization and casting for several months. At the time of cast removal, one month post-injury, excessive swelling of the wrist was noted; however, full digit mobility and cutaneous wrinkles were appreciated. A venous ultrasound revealed no signs of deep venous thrombosis. Six weeks later, examination revealed increased swelling and stiffness and a considerably decreased range of motion (ROM) of the right wrist and digits. Cutaneous exam showed sclerotic, mildly edematous, shiny skin with loss of wrinkles, involving all five digits (sclerodactyly), and the right dorsal and palmar hand and wrist, extending to the distal forearm (Figure 1). 

**Figure 2:**
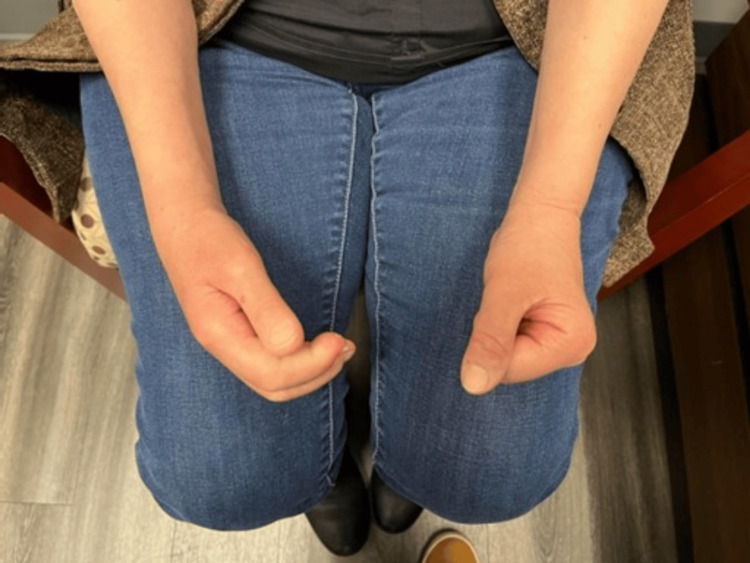
Unilateral sclerodactyly The patient was unable to make a closed fist with the right hand - prominent sclerodactyly is noted, which is not appreciated on the left hand.

**Figure 3:**
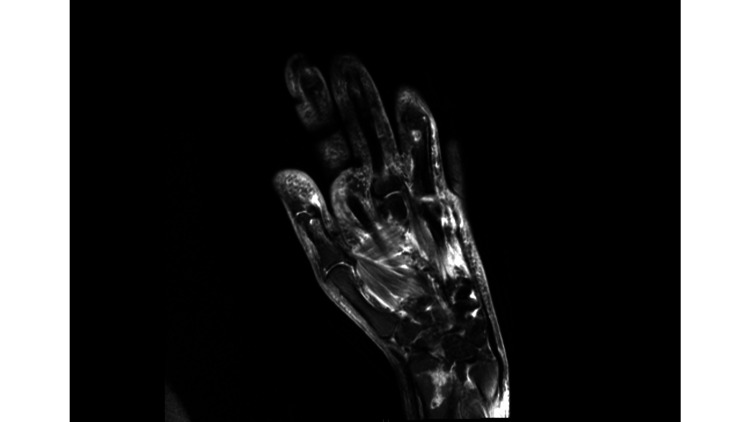
MRI of the right wrist without contrast Seen in this image is a comminuted intra-articular distal radial fracture with surrounding marrow edema along the fracture margins. The intramuscular signal is grossly normal without evidence of fluid collection, which suggests that a soft-tissue infection or hematoma is unlikely to be the cause of the patient's persistent wrist swelling.

Extension and flexion of the right wrist and digits were significantly decreased compared to the contralateral extremity, with an inability to make a closed fist (Figure 2). MRI showed a comminuted intra-articular distal radial fracture with surrounding marrow edema without evident signs of cellulitis or hematoma, ruling out infection or fluid collection as the cause of the persistent swelling (Figure 3).

**Figure 4:**
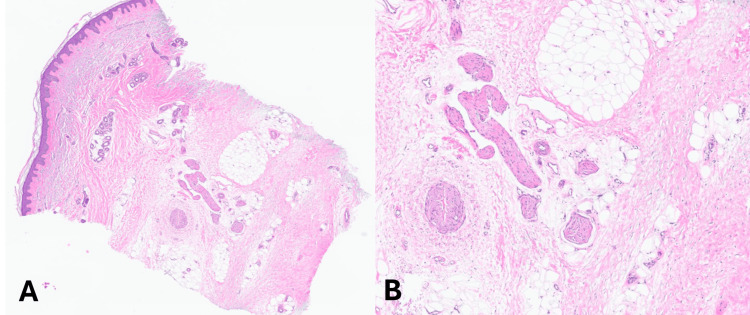
Histopathology showing scleroderma A) Hematoxylin and eosin (H&E) stain of skin biopsy at low power demonstrates homogenization of collagen at the base of the specimen, which is consonant with scleroderma. B) Higher power reveals a sparse lymphocytic infiltrate and mild fibrosis, which can be secondary to trauma. The lack of pronounced fibroblastic proliferation makes other sclerotic disorders such as scleromyxedema unlikely.

The patient was started on oral prednisone 30 mg daily, which mildly reduced swelling; however, she was unable to tolerate this dose. Upon tapering the dose, the skin tightness and decreased ROM persisted. A 4-mm punch biopsy showed homogenization of collagen in the lower portion of the biopsy, consistent with scleroderma, and it lacked pronounced fibroblastic proliferation seen in other sclerotic disorders such as scleromyxedema (Figure 4A, 4B).

Prednisone was tapered off, and the patient declined further immunosuppressive treatments. Follow-up examination at six months revealed no changes in findings.

## Discussion

LcSScl most commonly presents with bilateral symptoms and the presence of RP, nailfold capillary dilation, and sclerodactyly [1]. Unilateral skin changes, which are often present in morphea, are not characteristic of SScl. Although both disorders are thought to follow similar sclerosing pathways, current literature varies in reports of inducing etiologies. External factors such as trauma and radiation are associated with provoking morphea, yet are scarcely reported to induce SScl. In 1996, five cases of SScl following episodes of physical trauma were described [5] and another in 2001 [6]. In both reports, previously healthy patients suffered diffuse bilateral skin changes and internal organ involvement. However, unilateral progression of SScl, such as our case, appears to be an even rarer phenomenon.

Four cases have reported unilateral presentation of SScl following various insults, including ischemia [2], limb immobilization [3], and frequent exposure to hypothermic environments [4] (Table 1). Currently, the recommended management for cutaneous progression in SScl involves initiating systemic immunomodulators, such as mycophenolate mofetil or methotrexate, to halt activated inflammatory and fibrotic pathways. Of the cases of unilateral SScl, only one reported resolution of cutaneous symptoms following revascularization of the previously ischemic limb [2], suggesting that early reversal of the inciting inflammatory trigger may be a critical component of management. 

**Table 1:** Clinical presentation and associated inciting event for reports of unilateral systemic sclerosis RP = Raynaud's phenomenon

Type of Trauma	Article	Cases	Age/Gender	History of systemic sclerosis	Inciting Event	Time from trauma to onset of symptoms	Initial Presentation	Management and outcomes
Limb immobilization	Varga J [3]	2	47-year-old female	Bilateral RP for a few months	Immobilization due to nerve destruction resulting in right arm paralysis	3 weeks	Unilateral sclerosis and calcinosis cutis involving the right arm.	Treatment: Oral prednisone. Outcome: Progressed to complete aperistalsis of the distal esophagus and increased severity of bilateral RP
			40-year-old female	10-year history of bilateral RP	Left humerus fracture with casting for 1 month	1 month	Unilateral sclerosis, sclerodactyly, and decreased range of motion of the left upper extremity.	Treatment: Oral prednisone. Outcomes: No change in presentation
Ischemic injury	Roberts H [2]	1	55-year-old male	No	An arteriovenous fistula for haemodialysis was formed on the right arm	1 year	RP, sclerosis, sclerodactyly, and cutaneous ulcers confined to the right hand. The patient was also unable to form a fist and had reduced capillary refill compared to the contralateral hand.	Treatment: Distal revascularization of the affected limb was performed 5 months after the onset of symptoms. Outcomes: Complete resolution of all cutaneous symptoms
Hypothermic environment	Kubo S [4]	1	30-year-old female	No	Chronic exposure to a hypothermic environment (scooping ice cream) isolated to the right hand	6 years	RP, sclerosis, and nailfold capillary dilation and hemorrhage confined to the right hand	Not reported

The exact pathogenic pathway for SScl remains elusive but is believed to be a multifactorial process involving genetic predisposition and environmental triggers. It is postulated that vascular injury activates a wound healing cascade and production of profibrotic cytokines, such as transforming growth factor beta (TGF-β) and Wnt, resulting in excessive collagen deposition [1]. The role of trauma as a “triggering event” has been theorized to “unmask” the synthetic capacity of involved fibroblasts via direct blood vessel damage that causes a disturbed local microcirculation [6] and may explain the unilateral presentation in our case.

Other types of mechanical injury leading to neuronal deficits, like manual vibration and immobilization, reduce axon-reflex vasodilator responses, which may explain its causative link to RP and sclerodactyly confined to the area of effect, as seen in our case [7]. Such precipitating factors are more frequently associated with morphea, and although coexistence with SScl occurs [8], the presence of sclerodactyly eliminates morphea as a diagnosis here. Complex regional pain syndrome may demonstrate similar skin changes following physical trauma [9]; however, the lack of vasomotor instability, disproportionate pain, or radiologic signs of osteopenia render this an unlikely diagnosis.

Physical exertion has been reported as a possible precipitating factor for eosinophilic fasciitis (EF) [10], which bears some clinical and histologic similarities to SScl. The absence of peripheral eosinophilia, riverbed sign, and peau d’orange changes and the presence of sclerodactyly rule out EF as the diagnosis [11]. Herein, we report a case of unilateral progression of LcSScl after injury and immobilization, both for clinical interest and to highlight the potential role of trauma and immobilization in the pathogenesis of SScl.

## Conclusions

Herein, we report a case of trauma-induced unilateral progression of LcSScl following a radial fracture of the affected limb. This case highlights how focal injury and vascular disruption may activate the pathogenic mechanisms associated with SScl, resulting in symptoms localized to the affected area. Currently, there exists no definitive treatment for SScl, and further investigation into potential inducing etiologies may advance our understanding and management of the disease.
